# Chlorogenic Acid Inhibits Liver Fibrosis by Blocking the miR-21-Regulated TGF-β1/Smad7 Signaling Pathway *in Vitro* and *in Vivo*

**DOI:** 10.3389/fphar.2017.00929

**Published:** 2017-12-19

**Authors:** Fan Yang, Lei Luo, Zhi-De Zhu, Xuan Zhou, Yao Wang, Juan Xue, Juan Zhang, Xin Cai, Zhi-Lin Chen, Qian Ma, Yun-Fei Chen, Yu-Jie Wang, Ying-Ying Luo, Pan Liu, Lei Zhao

**Affiliations:** ^1^Department of Hepatology, Hubei Provincial Hospital of Traditional Chinese Medicine, Wuhan, China; ^2^School of Clinical Medical, Hubei University of Chinese Medicine, Wuhan, China; ^3^Guangxi University of Chinese Medicine, Nanning, China; ^4^Department of Infectious Diseases, Union Hospital, Tongji Medical College, Huazhong University of Science and Technology, Wuhan, China; ^5^Department of Gastroenterology, Hubei Provincial Hospital of Traditional Chinese and Western Medicine, Wuhan, China; ^6^Department of Pulmonary Diseases, Jingmen City Hospital of Traditional Chinese Medicine, Jingmen, China; ^7^School of Life Sciences, Hubei University, Wuhan, China; ^8^Department of Vascular Surgery, Union Hospital, Tongji Medical College, Huazhong University of Science and Technology, Wuhan, China; ^9^Department of Integrated Chinese and Western Medicine, Union Hospital, Tongji Medical College, Huazhong University of Science and Technology, Wuhan, China

**Keywords:** chlorogenic acid, liver fibrosis, TGF-β1, miR-21, Smad7

## Abstract

**Aims:** Chlorogenic acid (CGA) is a phenolic acid that has a wide range of pharmacological effects. However, the protective effects and mechanisms of CGA on liver fibrosis are not clear. This study explored the effects of CGA on miR-21-regulated TGF-β1/Smad7 liver fibrosis in the hepatic stellate LX2 cell line and in CCl4-induced liver fibrosis in Sprague-Dawley rats.

**Methods:** The mRNA expression of miR-21, Smad7, connective tissue growth factor (CTGF), α-smooth muscle actin (α-SMA), tissue inhibitor of metalloproteinase 1 (TIMP-1), matrix metalloproteinase-9 (MMP-9), and transforming growth factor-β1 (TGF-β1) and the protein levels of Smad2, p-Smad2, Smad3, p-Smad3, Smad2/3, p-Smad2/3, Smad7, CTGF, α-SMA, TIMP-1, MMP-9 and TGF-β1 were assayed in LX2 cells and liver tissue. The effects of CGA after miR-21 knockdown or overexpression were analyzed in LX2 cells. The liver tissue and serum were collected for histopathological examination, immunohistochemistry (IHC) and ELISA.

**Results:** The mRNA expression of miR-21, CTGF, α-SMA, TIMP-1, and TGF-β1 and the protein expression of p-Smad2, p-Smad3, p-Smad2/3, CTGF, α-SMA, TIMP-1, and TGF-β1 were inhibited by CGA both *in vitro* and *in vivo*. Meanwhile, CGA elevated the mRNA and protein expression of Smad7 and MMP-9. After miR-21 knockdown and overexpression, the downstream molecules also changed accordingly. CGA also lessened the degree of liver fibrosis in the pathological manifestation and reduced α-SMA and collagen I expression in liver tissue and TGF-β1 in serum. **Conclusion:** CGA might relieve liver fibrosis through the miR-21-regulated TGF-β1/Smad7 signaling pathway, which suggests that CGA might be a new anti-fibrosis agent that improves liver fibrosis.

## Introduction

Liver fibrosis is a chronic damage process to the liver characterized by the activation of hepatic stellate cells (HSCs), excessive accumulation of extracellular matrix (ECM) and distortion of hepatic architecture (Friedman, 2003; Bataller and Brenner, 2005; Gu et al., 2016). It is an important link in the further progression of hepatic cirrhosis, liver failure, and hepatocellular carcinoma (Iredale, 2007; Schuppan and Kim, 2013; Zhang et al., 2015a). Epidemiology has shown that some 45% of the deaths in developed countries are due to fibrotic diseases (Zhang et al., 2015b). In recent years, although many important studies have given us a better understanding of liver fibrosis (Luedde and Schwabe, 2011; Yan et al., 2011), no drug has been identified to have a definite effect against liver fibrosis. Therefore, searching for and developing efficient and well-tolerated drugs that can prevent progression toward liver fibrosis is urgently needed.

Transforming growth factor β1 (TGF-β1) is a necessary mediator in many fields such as the immune response, inflammation, matrix synthesis, cell growth, apoptosis, and differentiation (Inagaki and Okazaki, 2007; Saito et al., 2013; Xu et al., 2016). More importantly, TGF-β1 is a main profibrotic cytokines involved in the process of liver fibrosis (Cui et al., 2010), and the signaling pathway of TGF-β-Smad is an important signal transduction pathway in hepatic fibrosis. Meanwhile, several related studies have also confirmed that the inhibition of TGF-β1 expression and the regulation of the TGF-β-Smad signaling pathway are effective methods for the prevention of liver fibrosis (Bai et al., 2016). It is now clear that TGF-β1 can activate its downstream signaling pathway (Smad 2/3) to mediate fibrosis through binding to receptors on HSCs. In this signaling pathway, microRNA-21 (miR-21) positively regulates the production of collagen via Smad2/3 phosphorylation and is negatively regulated by Smad7 (Wells, 2000; Ikushima and Miyazono, 2012).

Chlorogenic acid (CGA, 5-O-caffeoylquinic acid), one of the most plentiful phenolic acids in nature, is formed by the esterification of quinic and caffeic acids (Suzuki et al., 2006), and is widely found in fruits, plants, and vegetables (Clifford, 1999), such as coffee beans (Bhattacharyya et al., 2014), honeysuckle (Luo et al., 2011), tobacco leaves (Niggeweg et al., 2004), and kiwi fruit (Li et al., 2014). Many researches have confirmed that CGA has multiple pharmacological effects, including anti-inflammatory (Shin et al., 2015), anti-hypertensive (Onakpoya et al., 2015), anti-oxidant capacities (Monteiro et al., 2007). CGA showed an anti-hepatotoxic effect on LPS-treated mice by suppressing the levels of TLR4 and the NF-κB p65 subunit (Xu et al., 2010) and an anti-fibrosis effect on DMN-induced liver fibrosis in rats (Shi et al., 2016). We have determined that CGA has an anti-liver fibrosis effect on schistosome-infected mice by suppressing the IL-13/miR-21/Smad7 signaling interactions (Wang et al., 2017) and has an anti-inflammatory effect through the suppression of the TLR2/TLR9-Myd88 signaling pathways (Guo et al., 2015a).

However, whether CGA can inhibit liver fibrosis through the miR-21-regulated TGF-β1/Smad7 signaling pathway has not been studied. Therefore, in this research we investigated the therapeutic effect and mechanisms of CGA on anti-fibrosis by interacting with the miR-21-regulated TGF-β1/Smad7 signaling pathway in CCl4-induced liver fibrosis rat model and TGF-β1-stimulated human HSC line LX-2.

## Materials and Methods

### Reagents and Antibodies

Chlorogenic acid (≥95% titration) was obtained from Sigma–Aldrich China (Shanghai, China). Human recombinant TGF-β1was purchased from Peprotech (Rocky Hill, NJ, United States). A rat TGF-β1 ELISA kit was purchased from Elabscience Biotechnology Co., Ltd. (Wuhan, China). Foetal bovine serum (FBS) and RPMI 1640 basic were purchased from Gibco (Grand Island, NY, United States). Cell counting kit-8 (CCK-8) was purchased from Dojindo Company (Japan). CCl4 was purchased from Sigma (St. Louis, MO, United States). Rabbit anti-rat Smad2, p-Smad2, Smad3, p-Smad3, Smad2/3, p-Smad2/3 and TGF-β1 antibodies were purchased from Cell Signaling Technology (CST, Boston, MA, United States). Smad7, CTGF, α-SMA, MMP-9, TIMP-1, collagen I, glyceraldehyde-3-phosphate dehydrogenase (GAPDH) and horseradish peroxidase (HRP)-labeled secondary antibody were acquired from Wuhan Boster Biotechnology Co., Ltd. (Wuhan, China). The Trizol reagent, PeproTech RNAiso Plus and realtime polymerase chain reaction (PCR) kit were purchased from TaKaRa (Dalian, China). The miR-21 and negative control lentiviral vectors were synthesized by Gene Chem Co., Ltd. (Shanghai, China).

### Cell Culture and CGA Treatment

The LX2 cells were cultured in RPMI 1640 medium with 10% foetal bovine serum (FBS) at 37°C in 5% CO2 and 95% humidified air. Cell morphological changes after treatments were observed by a regular phase contrast microscope. Real-time PCR and western blot analysis were employed to evaluate the effect of CGA on LX2 cells. The cells were passaged in 6-well plates overnight and pretreated with CGA at various concentrations (20 μg/ml, 40 μg/ml and 80 μg/ml) for 24 h. For the last 6 h, TGF-β1 (10 ng/ml) was added to the wells for modeling but not to those for the normal group. After 24 h, the supernatants and cells were collected.

### Cell Cytotoxic Assays

The procedures were carried out according to our past study (Li et al., 2016). The cell counting kit-8 (CCK-8) assay was employed to examine cell cytotoxicity. LX2 cells were seeded in 96-well plates at a density of 5.0 × 10^4^ cells/ml. CGA at different concentrations (20 μg/ml, 40 μg/ml and 80 μg/ml) was added. After 24 h, CCK-8 with 10 μl solution was added and the cells were incubated at 37°C for 2 h before meaturing the absorbance at 450 nm on a microplate reader. The experiments were repeated in duplicate.

### Lentivirus-Mediated miR-21 Overexpression or Knockdown

To over-express the miR-21 gene, GV273-miR-21/NC-EGFP was transfected into the 293 T cell line. Viral supernatant were collected from the transfected 293 T cells after 48 h (3 × 10^8^ TU/ml) and were used to infect the LX2 cells. To knockdown miR-21 expression, the specific sequence of hsa-miR-21-3p-inhibition (16129-1) was 5′-TCGAGAAAAAACAGCCCATCGACTGGTGTTGTTTTTC-3′, and the reference sequence was 5′-TTCTCCGAACGTGTCACGT-3′. GV369-miR-21/NC-EGFP was transfected into the 293T cell line, and the viral supernatant was collected after 48 h (5 × 10^8^ TU/ml). LX2 cells were transfected with lentivirus at a multiplicity of infection (MOI) of 10 plaque-forming units (PFU)/cell according to the manual. The medium was replaced 12 h later, and then the cells continued to be incubated for 72 h. overexpression and knockdown of miR-21 was confirmed by Real-time PCR analysis.

### Animal Groups

Fifty male Sprague-Dawley rats (180 to 220 g) were obtained from the Hubei Provincial Centers for Disease Control and Prevention (Wuhan, China). All rats were housed in a room with controlled temperature (22–25°C), a 12:12 h light-dark cycle and free access to water and food. All study protocols abided by internationally accepted principles and the Guidelines for the Care and Use of Laboratory Animals of Huazhong University of Science and Technology and was approved by the Ethics Committee of Union Hospital, Tongji Medical College, Huazhong University of Science and Technology. The rats were randomly divided into 5 groups (*n* = 10): CGA low dosage (15 mg/kg), CGA middle dosage (30 mg/kg), CGA high dosage (60 mg/kg), and the experiment and normal groups. All groups except the normal group were given a CCl4 intra-peritoneal injection (i.p.) to induce liver fibrosis (CCl4: olive oil, 2:3 (vol/vol) per kg body weight, 4 ml/kg for the first dose and then 2 ml/kg twice per week) for 8 weeks. After 4 weeks, the rats in the low, middle, and high-dose CGA groups were administered CGA at 15 mg/kg, 30 mg/kg, or 60 mg/kg (at a concentration of 1.5, 3, or 6 mg/ml, the feeding volume was 1 ml/100 g) for four consecutive weeks, and the rats in the experiment and normal groups were administered normal saline at the same volume of 1 ml/100 g.

### Liver Histopathological Evaluation

The rats were sacrificed to collect specimens. The procedure adhered to our past experiments (Huang et al., 2013; Jin et al., 2015). The liver tissues were collected and fixed in 4% paraformaldehyde solution, embedded in paraffin for histological examinations and stained with haematoxylin-eosin (HE) and Masson’s trichrome to assess liver damage and fibrosis development. Histological changes were observed at magnifications of ×100 and ×200. Five non-consecutive and random histological fields were analyzed to obtain the mean value.

### Enzyme-Linked Immunosorbent Assay (ELISA) for Measuring TGF-β1 Expression in Serum

The procedures were referred to in our previous study (Du et al., 2016). Expression of TGF-β1 in serum was determined by sandwich ELISA. The serum was collected and assayed for TGF-β1 by a rat TGF-β1 ELISA kit. The procedure conformed to the directions in the instruction manual from the kit.

### Quantitative Real-Time PCR for Detecting mRNA Expression

The procedures were carried out according to our previous study (Zhou et al., 2013; Li et al., 2017). Total RNA was extracted from LX2 cells and liver tissue using TRIzol reagent (Invitrogen) and the cDNA was produced by using a PrimeScript^TM^ RT reagent kit according to the manufacturer’s protocol. Real-time qPCR was carried out in a 10 μl reaction containing 0.2 μl of cDNA and incubated at 37°C for 15 min and 85°C for 5 s. Real-time PCR reactions were performed at 95°C for 10 s, followed by 40 cycles of 95°C for 5 s and 60°C for 20 s according to the instructions of the SYBR Premix Ex Taq kit. The data were analyzed by the 2^-ΔΔC_T_^ method. All primers were synthesized by GenScript (Nanjing, China), and the sequences are shown as **Table 1**.

**Table 1:** Primer sequences for Real-time PCR.

Target genes	Primer sequence	Human (5′→3′)	Rat (5′→3′)
miR-21	RT stem-loop	CTCAACTGGTGTCGTGGAGTCGGCAATTCAGTTGAGTCAACATC	GTCGTATCCAGTGCAGGGTCCGAGGTATTCGCACTGGATACGACGACAGC
	Forward primer	ACACTCCAGCTGGGTAGCTTATCAGACTGA	GCCGAGCAACAGCAGTCGAT
	Reverse primer	TGGTGTCGTGGAGTCG	CAGTGCAGGGTCCGAGGTAT
Smad7	Forward primer	AGAGGCTGTGTTGCTGTGAAT	GCTGTACCTTCCTCCGATGA
	Reverse primer	GCAGAGTCGGCTAAGGTGATG	CAAAAGCCATTCCCCTGAGG
CTGF	Forward primer	AATGCTGCGAGGAGTGGGT	GGCAGGGCCAACCACTGTGC
	Reverse primer	CGGCTCTAATCATAGTTGGGTCT	CAGTGCACTTGCCTGGATGG
α-SMA	Forward primer	GACAGCTACGTGGGTGACGAA	AGAAGCCCAGCCAGTCGCCATCA
	Reverse primer	CGGGTACTTCAGGGTCAGGAT	AGCAAAGCCCGCCTTACAGAGCC
MMP-9	Forward primer	TGTACCGCTATGGTTACACTCG	AAAGGTCGCTCGGATGGTTAT
	Reverse primer	GGCAGGGACAGTTGCTTCT	CTGCTTGCCCAGGAAGACGAA
TIMP-1	Forward primer	ACCACCTTATACCAGCGTTA	AGCCCTGCTCAGCAAAAGG
	Reverse primer	AAACAGGGAAACACTGTGCA	CTG TCC ACA AGC AAT GAC TGT CA
TGF-β1	Forward primer		CCCCTGGAAAGGGCTCAACAC
	Reverse primer		TCCAACCCAGGTCCTTCCTAAAGTC
GAPDH	Forward primer	ACCACAGTCCATGCCATCAC	GGCACAGTCAAGGCTGAGAATG
	Reverse primer	TCCACCACCCTGTTGCTGTA	ATGGTGGTGAAGACGCCAGTA

### Western Blot Analysis

The procedures followed our past experimental steps (Guo et al., 2015b; Ding et al., 2016). For liver tissue lysate preparation, liver tissue was homogenized with a hand-held homogenizer in lysis buffer. For cell lysate preparation, the LX2 monolayer cells were rinsed with PBS and lysed in RIPA buffer with a cocktail of protease inhibitors on ice. Total protein was extracted from the liver tissue and LX2 cells. The protein concentration was determined with a BCA protein assay kit. In each protein sample, an equivalent volume of 2× sodium dodecyl sulphate (SDS) loading buffer (100 mM Tris-HCl, pH 6.8; 4% SDS; 20% glycerine; 10% β-mercaptoethanol; and 0.2% bromophenol blue) was added and mixed again. The mixtures were then denatured at 95°C for 10 min, and the protein was loaded into each well and separated with 10% SDS-PAGE gel. After separation for approximately 80 min, the proteins were transferred to PVDF membranes, and the PVDF membranes were saturated with 5% non-fat milk containing in PBS for 1 h at room temperature and then probed with specific antibodies overnight at 4°C. The membrane was washed after incubation and then incubated for 1 h with the HRP-labeled secondary antibody. After further washing the membrane with TBST three times, bands were identified with enhanced chemiluminescence (ECL) reagent, and the signals were detected by exposing the membranes to X-ray films. The dilutions of the primary and secondary antibody were as follows: Smad2, 1:1000; Smad3, 1:1000; Smad2/3, 1:1000; p-Smad2, 1:1000; p-Smad3, 1:1000; p-Smad2/3, 1:1000; TGF-β1, 1:500; α-SMA, 1:1000; Smad7, 1:1000; CTGF, 1:800; MMP-9, 1:1000; TIMP-1, 1:1000; GAPDH, 1:5000; and secondary antibody 1: 5000. The Fuji ultrasonic-Doppler velocity profile (UVP) system and the ImageJ program were used for the densitometry analysis of the immunoreactive bands.

### Immunohistochemistry (IHC) for Detection of α-SMA and Collagen I Expression in Liver Tissue

The procedure adhered to our previous study (Yang et al., 2016). The liver tissue (10 μm) were deparaffinized and hydrated, then the soaked slides were inactivated in 5% H2O2/methanol to block endogenous peroxidase activity. Next, the slides were incubated with normal goat serum for 10 min and incubated with α-SMA antibody (dilution, 1:500) or collagen I antibody (dilution, 1:200) at 4°C overnight. The slides were washed the next day and incubated with biotinylated secondary antibody at 37°C for 60 min. Then, the slides were washed again and incubated with horseradish peroxidase-labeled streptavidin at 37°C. The samples were developed with diaminobenzidine (DAB) and stained with haematoxylin. After the slides were washed with distilled water and dehydrated, they were made transparent and mounted under a microscope for examination. Image-Pro Plus software was employed to evaluate the mean optical density value of the images after immunohistochemical analysis.

### Statistical Analysis

The results are reported as the mean ± standard deviation (SD). Comparisons of the data between groups were expressed with Student’s *t*-test and one-way ANOVA followed by Tukey’s test. Statistical significance was considered significant when the *P*-value < 0.05 (Jin et al., 2013; Ding et al., 2015). All statistical analyses were performed with SPSS 12.0. Graphs were drawed with GraphPad Prism software (version 6).

## Results

### Cytotoxicity of CGA on LX2 Cells

**FIGURE 1:**
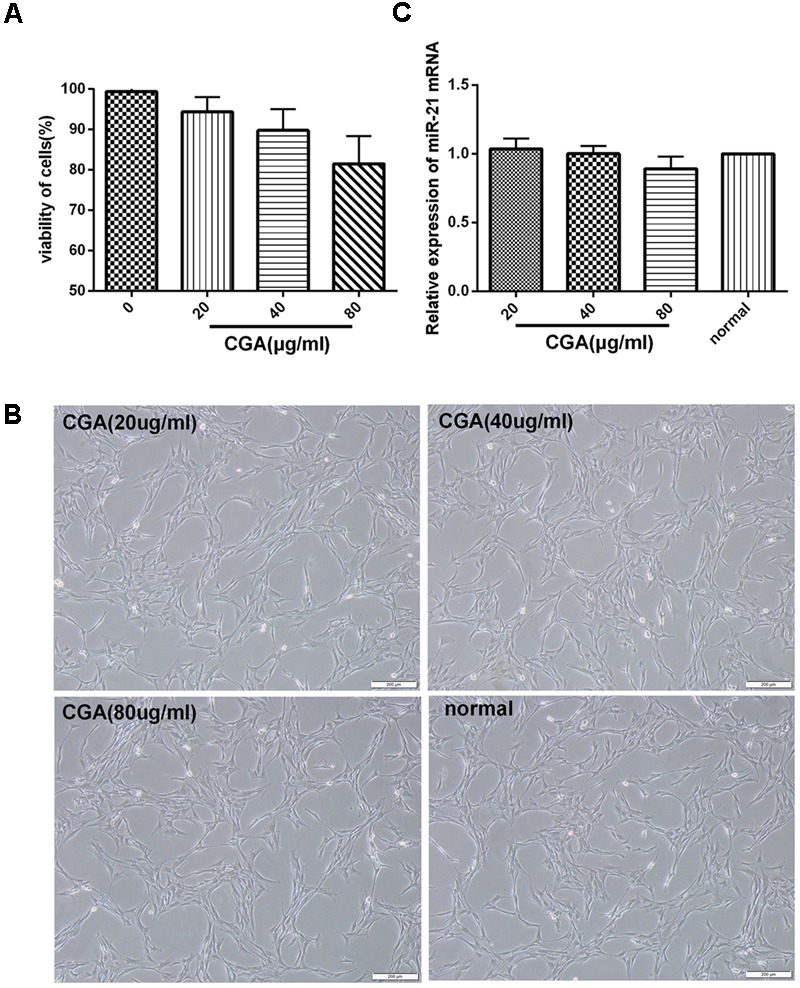
Cytotoxicity of CGA on LX2 cells was determined by CCK8 assay. **(A)** LX2 cells plated in 96-well plates were treated with CGA for 24 h, and cell viability was determined. **(B)** Cell morphologies of LX2 after CGA treatment for 24 h. **(C)** The mRNA level of miR-21 was measured by quantitative real-time PCR.

CCK8 assay showed that pretreatment on unstimulated LX2 cells with a series of concentrations of CGA at 20 μg/ml, 40 μg/ml and 80 μg/ml for 24 h did not significantly affect cell viability (**Figure 1A**). The same result was shown for the cell morphology observation (**Figure 1B**). In addition, when assayed with real-time PCR, the difference of changes of miR-21 between CGA-treated cells and the normal group was not significant (**Figure 1C**). Therefore, we chose CGA at 20 μg/ml, 40 μg/ml, and 80 μg/ml to treat LX2 cells for 24 h.

### TGF-β1 Induces miR-21 and CTGF Expression in LX2 Cells

**FIGURE 2:**
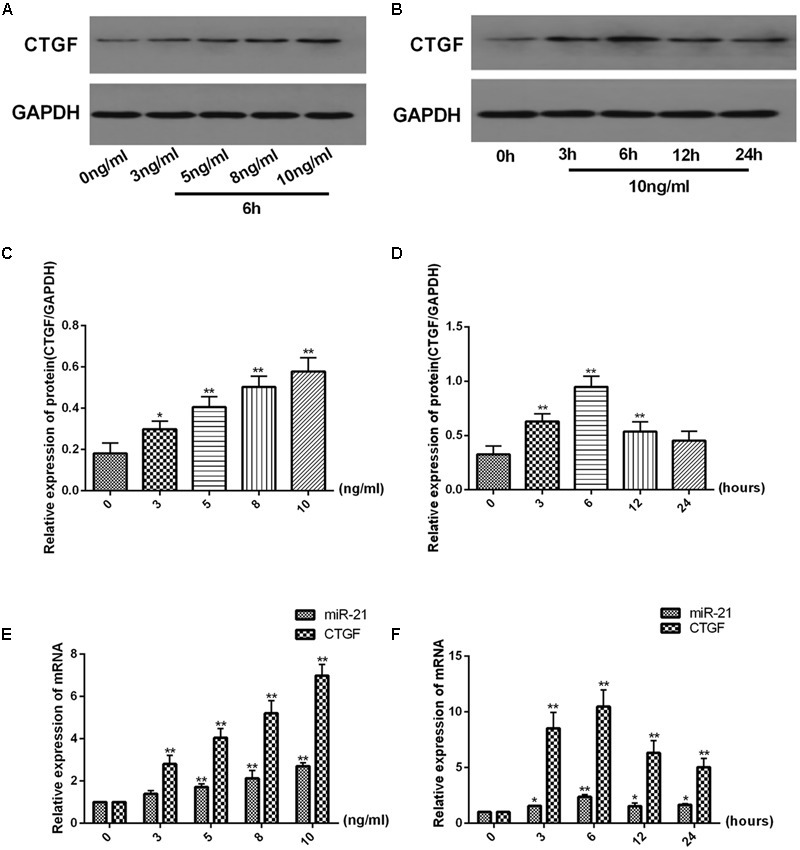
TGF-β1 increases miR-21 and CTGF expression in a dose-dependent manner and decreases it in a time-dependent manner. **(A,B)** The protein levels of CTGF were measured by western blot. **(C,D)** The ratio of CTGF to GAPDH was obtained by densitometric analysis. **(E,F)** The mRNA levels of miR-21 and CTGF were detected by real-time quantitative PCR. Data are shown as the means ± SD. ^∗^*P* < 0.05, ^∗∗^*P* < 0.01. compared with either time point 0 or the untreated group.

There was a dose-dependent increase and time-dependent change in the protein expression of CTGF and in the mRNA expression of miR-21 and CTGF in LX2 cells treated with TGF-β1 (**Figure 2**). The protein expression of CTGF was measured after treatment with TGF-β1 at a range of 0–10 ng/ml for 6 h (**Figure 2A**) and treated with TGF-β1 at 10 ng/ml for 0–24 h in LX2 cells (**Figure 2B**). As shown in **Figure 2C**, the protein level of CTGF was significantly increase after treatment with TGF-β1 at 10 ng/ml compared with the 0 ng/ml group. In **Figure 2D**, when treated with 10 ng/ml TGF-β1 for 0–24 h. The protein expression of CTGF was highest at time point of 6 h compared with the 0 h group. The same result was shown for the mRNA expression of miR-21 and CTGF. As shown in **Figures 2E,F**, TGF-β1 treatment at 10 ng/ml for 6 h significantly increased the mRNA expression of miR-21 and CTGF (*P* < 0.01), so we chose TGF-β1 treatment at 10 ng/ml for 6 h for subsequent experiments.

### Effect of CGA on the miR-21-Regulated TGF-β1/Smad7 Signaling Pathway in LX2 Cells after TGF-β1 Stimulation

**FIGURE 3:**
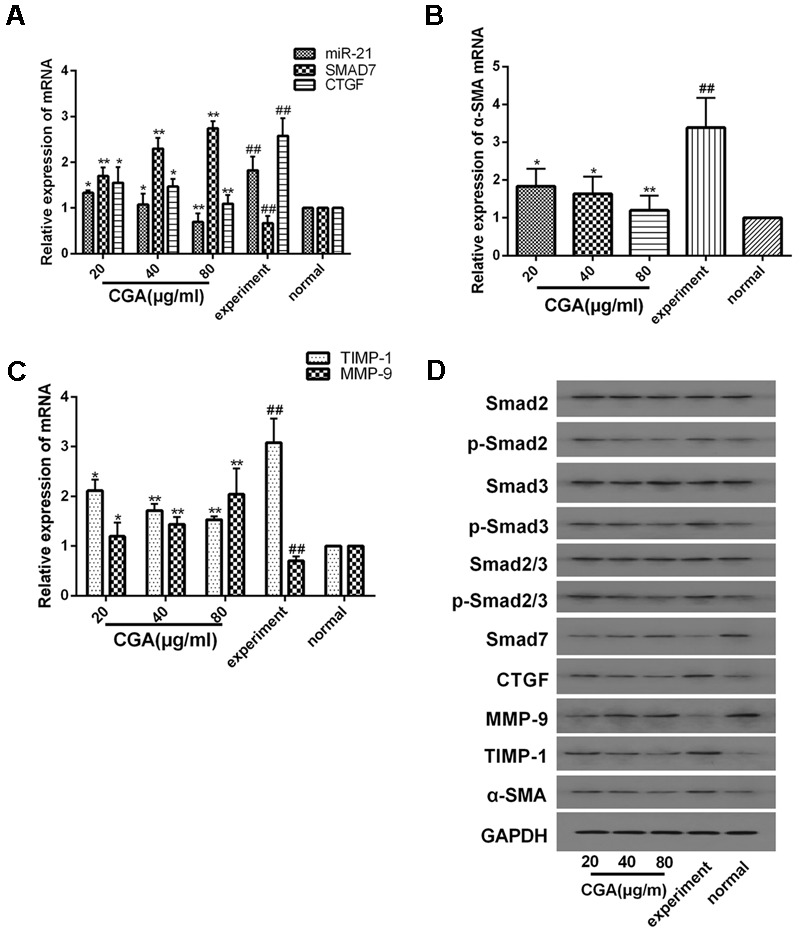
Effects of CGA on the TGF-β1/miR-21/Smad7 signaling pathway in LX2 cells after TGF-β1 stimulation. **(A–C)** The mRNA levels were detected by real-time quantitative PCR. **(D)** The protein levels were assayed by western blotting. The data from three independent experiments are expressed as the means ± SD. ^∗^*P* < 0.05 compared with the experimental group; ^∗∗^*P* < 0.01 compared with experimental group; ^##^*P* < 0.01 compared with the normal group.

As shown in **Figures 3A–C**, compared with the normal group, the mRNA levels of miR-21, CTGF, TIMP-1 and α-SMA in the experimental group were significantly increased (*P* < 0.01), and the mRNA levels of Smad7 and MMP-9 were notably decreased (*P* < 0.01). After treatment with CGA at a series of concentrations for 24 h, the mRNA levels of miR-21, CTGF, α-SMA and TIMP-1 were decreased, and the mRNA levels of Smad7 and MMP-9 were increased compared with the experimental group (*P* < 0.05 or 0.01). As shown in **Figure 3D**, western blot analysis was used to assess the protein levels. Compared with the normal group, the protein expression of CTGF, α-SMA, TIMP-1, p-Smad2, p-Smad3 and p-Smad2/3 were significantly elevated and the protein levels of Smad7 and MMP-9 were decreased in TGF-β1-stimulated LX2 cells. However, the protein expression of CTGF, α-SMA, TIMP-1, p-Smad2, p-Smad3 and p-Smad2/3 was effectively inhibited after treatment with CGA, and CGA could improve the protein expression of Smad7 and MMP-9 compared with the experimental group. The protein expression of Smad2, Smad3 and Smad2/3 had no significant changes.

### Verification of Downstream Signaling Molecules in LX2 Cells after miR-21 Overexpression

**FIGURE 4:**
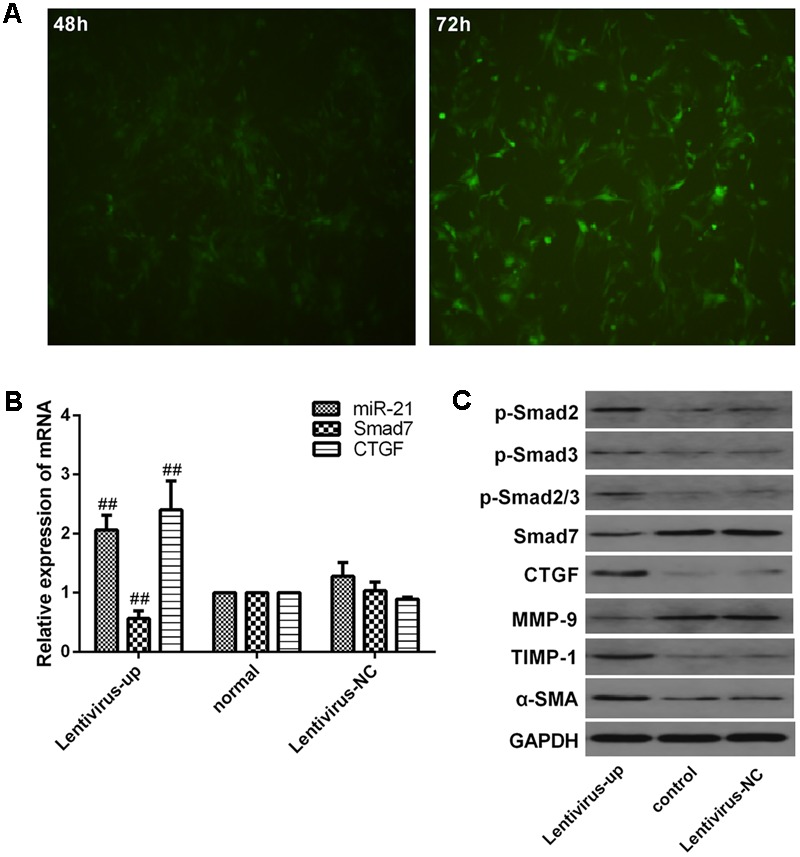
Verification of downstream signaling molecules in LX2 cells after miR-21 overexpression. **(A)** LX2 cells were transfected with lentivirus, and the expression of GFP was observed with a fluorescence microscope after 48 and 72 h. **(B)** The expression of miR-21, Smad7 and CTGF were measured by quantitative real-time PCR. **(C)** The protein expression was detected by western blotting. Data are shown means ± SD and significant differences were determined by one-way ANOVA. ^##^*P* < 0.01 for lentivirus-up group vs. normal group.

We investigated the expression of downstream signaling molecules after transfecting LX2 cells *in vitro* with the miR-21 lentiviral vector GV369. The green fluorescent protein (GFP) was observed with a fluorescence microscope after transfection for 48 and 72 h (**Figure 4A**). Meanwhile, to confirm the transduction efficiency, the mRNA expression of miR-21, Smad7 and CTGF, and the protein levels of p-Smad2, p-Smad3, p-Smad2/3, CTGF, Smad7, α-SMA, TIMP-1 and MMP-9 were detected at 72h after transfection (**Figures 4B,C**). We observed that there was no difference in the mRNA levels of miR-21, Smad7 and CTGF (*P* > 0.05, **Figure 4B**) and the protein levels of p-Smad2, p-Smad3, p-Smad2/3, CTGF, Smad7, α-SMA, TIMP-1 and MMP-9 between the normal and lentivirus-NC group (lentivirus negative control group) (*P* > 0.05, **Figure 4C**). However, compared with the normal group, after miR-21 overexpression, the mRNA expression of miR-21 and CTGF increased, and the mRNA expression of Smad7 decreased (*P* < 0.01, **Figure 4B**). The protein expression of p-Smad2, p-Smad3, p-Smad2/3, CTGF, α-SMA and TIMP-1 increased, and the protein levels of Smad7 and MMP-9 decreased (*P* < 0.01, **Figure 4C**).

### Effect of CGA on the miR-21-Regulated TGF-β1/Smad7 Signaling Pathway in LX2 Cells after miR-21 Overexpression

**FIGURE 5:**
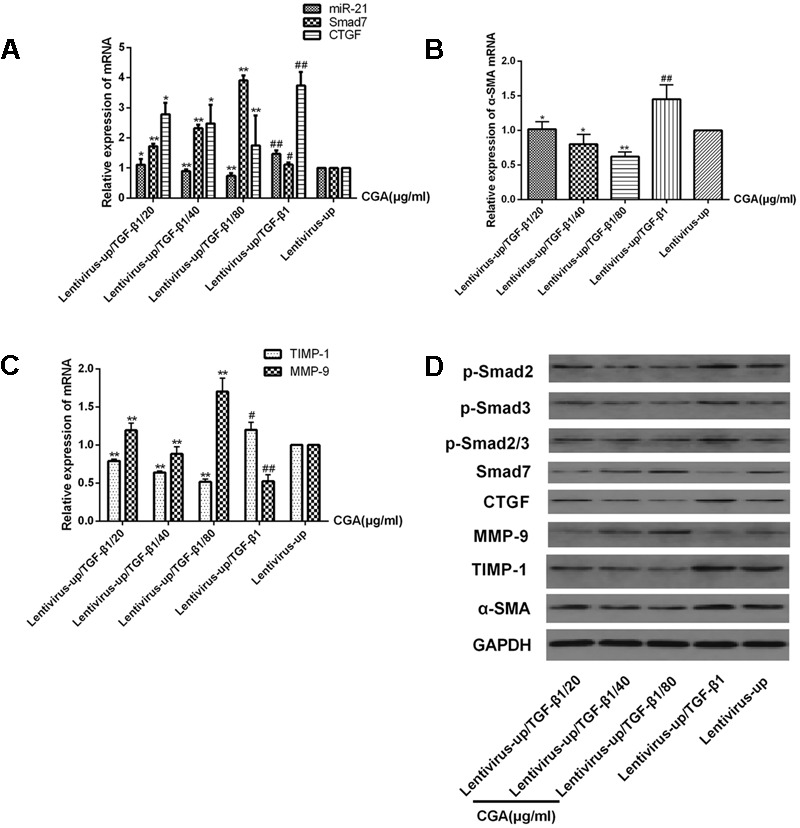
Effects of CGA on the TGF-β1/miR-21/Smad7 signaling pathway in LX2 cells after TGF-β1 overexpression. **(A–C)** The mRNA levels were detected by real-time quantitative PCR. **(D)** The protein levels were assayed by western blotting. The data from three independent experiments are expressed as the means ± SD. ^∗^*P* < 0.05 for lentivirus-up/TGF-β1/CGA vs. lentivirus-up/TGF-β1; ^∗∗^*P* < 0.01 for lentivirus-up/TGF-β1/CGA vs. lentivirus-up/TGF-β1; ^#^*P* < 0.05 compared with the normal group; ^##^*P* < 0.01 compared with the normal group.

The miR-21 in LX2 cells was overexpressed with the lentiviral vector for 72 h, and then, the cells were treated with a series of concentrations of CGA (20 μg/ml, 40 μg/ml, and 80 μg/ml) for 24 h. TGF-β1 was added to the LX2 cells for the last 6 h before harvest. As shown in **Figures 5A–C**, compared with the lentivirus-up group, the mRNA levels of miR-21, CTGF, α-SMA and TIMP-1 in the lentivirus-up/TGF-β1 group were increased (*P* < 0.01 or 0.05), and the mRNA levels of Smad7 and MMP-9 were decreased (*P* < 0.01 or 0.05). The protein expression of p-Smad2, p-Smad3, p-Smad2/3, CTGF, α-SMA and TIMP-1 were also increased, and the protein levels of Smad7 and MMP-9 were decreased (**Figure 5D**). After treatment with CGA at a series of concentrations, compared to that in lentivirus-up/TGF-β1 group, the mRNA levels of miR-21, CTGF, α-SMA and TIMP-1 were decreased (**Figures 5A–C**, *P* < 0.05 or 0.01), and the mRNA levels of Smad7 and MMP-9 were increased relatively (**Figures 5A–C**, *P* < 0.01 or 0.05). The protein levels of CTGF, α-SMA, TIMP-1, p-Smad2, p-Smad3, and p-Smad2/3 were decreased, and the protein levels of Smad7 and MMP-9 were increased (**Figure 5D**).

### Verification of Downstream Signaling Molecules in LX2 Cells after miR-21 Knockdown

**FIGURE 6:**
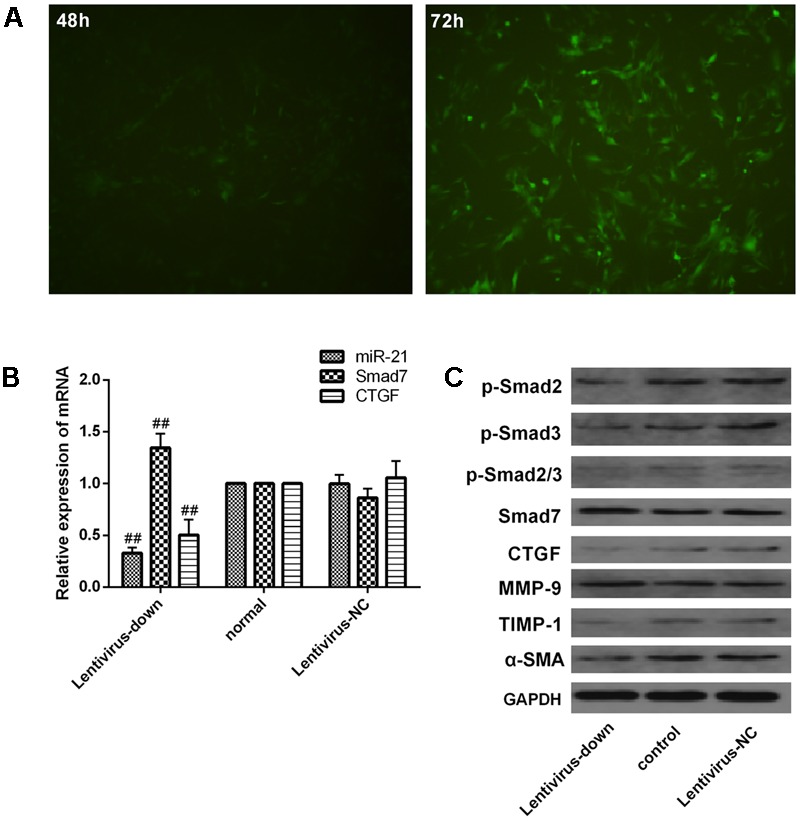
Verification of downstream signaling molecules in LX2 cells after miR-21 knockdown. **(A)** LX2 cells were transfected with lentivirus, and the expression of GFP was observed with a fluorescence microscope after 48 and 72 h. **(B)** The expression of miR-21, Smad7 and CTGF was measured by quantitative real-time PCR. **(C)** The protein expression was detected by western blotting. Data are shown as the means ± SD, and significant differences were determined by one-way ANOVA. ^##^*P* < 0.01 for lentivirus-down group vs. normal group.

We investigated the expression of downstream signaling molecules after transfecting LX2 cells *in vitro* with the miR-21 lentiviral vector GV273. The green fluorescent protein (GFP) was observed with a fluorescence microscope after transfection for 48 and 72 h (**Figure 6A**). Meanwhile, to confirm the transduction efficiency, the mRNA expression of miR-21, Smad7 and CTGF, and the protein levels of p-Smad2, p-Smad3, p-Smad2/3, CTGF, Smad7, α-SMA, TIMP-1 and MMP-9 were detected at 72h after transfection (**Figure 6B,C**). We observed that there was no difference between the normal and lentivirus-NC group (lentivirus negative control group) in the mRNA levels of miR-21, Smad7 and CTGF (**Figure 6B**, *P* > 0.05) and the protein levels of p-Smad2, p-Smad3, p-Smad2/3, CTGF, Smad7, α-SMA, TIMP-1 and MMP-9 (**Figure 6C**). However, compared with the normal group, after miR-21 knockdown, the mRNA expression of miR-21 and CTGF decreased, and the mRNA expression of Smad7 increased (*P* < 0.01, **Figure 6B**). The protein expression of p-Smad2, p-Smad3, p-Smad2/3, CTGF, α-SMA and TIMP-1 decreased, and the protein levels of Smad7 and MMP-9 increased (**Figure 6C**, *P* < 0.01).

### Effect of CGA on the miR-21-Regulated TGF-β1/Smad7 Signaling Pathway in LX2 Cells after TGF-β1 Knockdown

**FIGURE 7:**
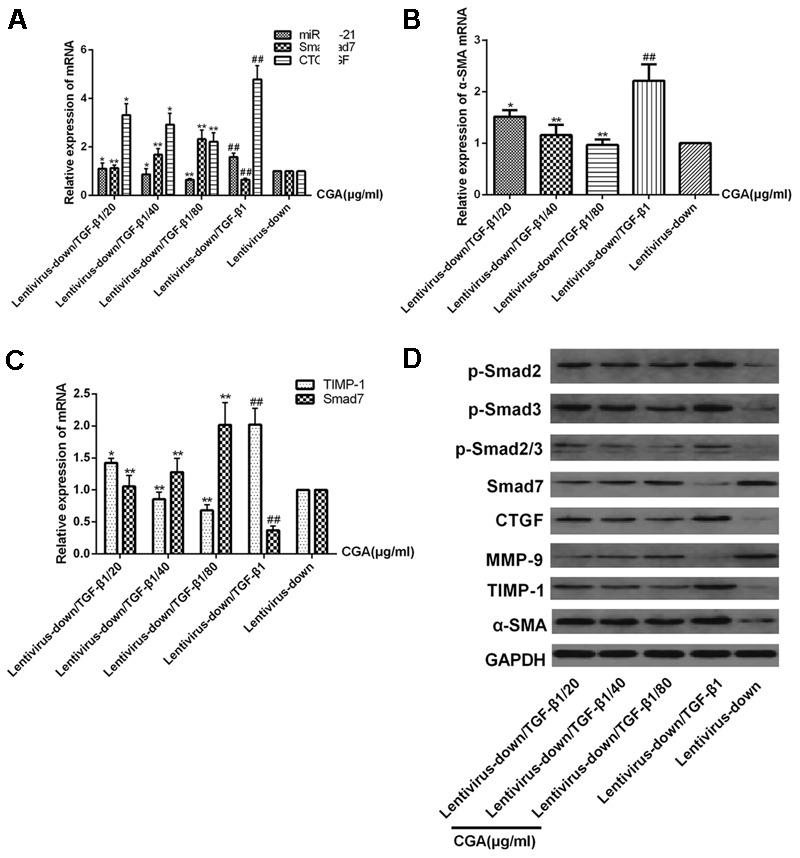
Effects of CGA on the TGF-β1/miR-21/Smad7 signaling pathway in LX2 cells after TGF-β1 knockdown. **(A–C)** The mRNA levels were detected by real-time quantitative PCR. **(D)** The protein levels were assayed by western blotting. The data from three independent experiments are expressed as the means ± SD. ^∗^*P* < 0.05 for lentivirus-down/TGF-β1/CGA vs. lentivirus-down/TGF-β1; ^∗∗^*P* < 0.01 for lentivirus-down/TGF-β1/CGA vs. lentivirus-down/TGF-β1; ^##^*P* < 0.01 lentivirus-down/TGF-β1 vs. lentivirus-down.

The miR-21 in LX2 cells was knocked-down with a lentiviral vector for 72 h, and then, the cells were treated with a series of concentrations of CGA (20 μg/ml, 40 μg/ml, and 80 μg/ml) for 24 h. TGF-β1 was added to the LX2 cells for the last 6 h before harvest. As shown in **Figures 7A–C**, compared with the lentivirus-down group, the mRNA levels of miR-21, CTGF, α-SMA and TIMP-1 in the lentivirus-down/TGF-β1 group were increased (*P* < 0.01), and the mRNA levels of Smad7 and MMP-9 were decreased (*P* < 0.01). The protein expression levels of p-Smad2, p-Smad3, p-Smad2/3, CTGF, α-SMA and TIMP-1 were also decreased, and the protein expression levels of Smad7 and MMP-9 were increased (**Figure 7D**). After treatment with CGA at a series of concentrations, the mRNA levels of miR-21, CTGF, α-SMA and TIMP-1 were decreased (**Figures 7A,B**, *P* < 0.05 or 0.01), and the mRNA levels of Smad7 and MMP-9 increased relatively (**Figure 7C**, *P* < 0.05 or 0.01) compared to that in the lentivirus-down/TGF-β1 group. The protein levels of CTGF, α-SMA, TIMP-1, p-Smad2, p-Smad3, and p-Smad2/3 were decreased, and the protein levels of Smad7 and MMP-9 were increased (**Figure 7D**).

### Effect of CGA on the Expression of TGF-β1 in Serum

**FIGURE 8:**
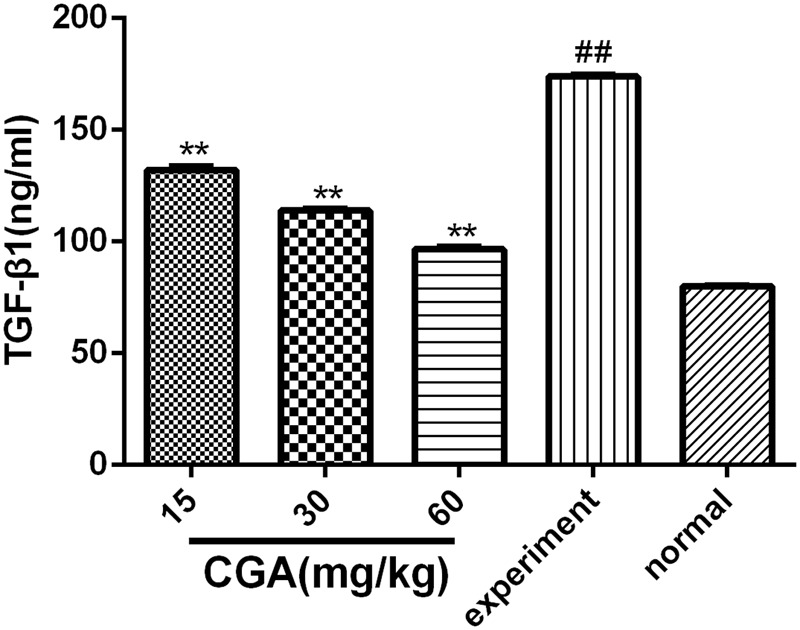
Effect of CGA on the expression of TGF-β1 in serum, and the level of TGF-β1 was determined by ELISA. Data are shown as the means ± standard deviation. *n* = 10, ^∗∗^*P* < 0.01 compared with the experimental group; ^##^*P* < 0.01 compared with the normal group, as determined by Student’s *t*-test.

As shown in **Figure 8**, the expression level of TGF-β1 in the serum in the experimental group was markedly increased when compared with that in the normal group (*P* < 0.01). The level of TGF-β1 was decreased significantly compared with that in the experimental group when the rats were treated with CGA at different concentrations (*P* < 0.05 or 0.01). The inhibitory effect was enhanced when the concentration of CGA increased (*P* < 0.01), which suggested that CGA could inhibit the expression of TGF-β1.

### Effect of CGA on Liver Histopathology

**FIGURE 9:**
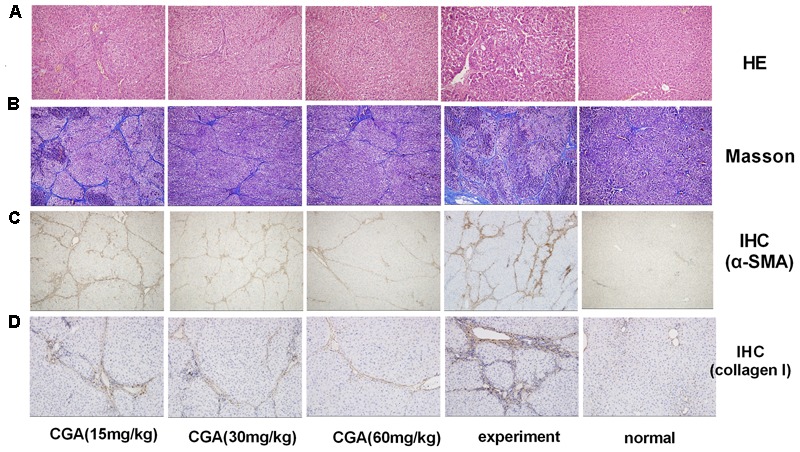
Evaluation of the effect of CGA on liver histopathological and immunohistochemistry (IHC) in liver tissue. **(A)** Histological images of rat livers stained with H&E (original magnification, ×200). **(B)** The histopathologic detection of collagen in the liver by Masson’s trichrome stain (original magnification, ×100). **(C,D)** Effects of CGA on α-SMA and collagen I expression were examined with immunohistochemistry in liver tissue (original magnification, ×100).

Haematoxylin-eosin and Masson’s trichrome staining were used to evaluate the anti-fibrosis role of CGA. As shown in **Figure 9A**, slices from the liver of the normal group showed liver structural integrity without inflammatory cell infiltration. However, CCl4-induced liver injury in the experimental group showed fibrosis and inflammatory cell infiltration and the loss of structural integrity. Nevertheless, in the CGA group, the slices showed less fibrosis, less inflammatory cell infiltration and less liver cell necrosis compared with the experimental group. As shown in **Figure 9B**, collagen is blue in fibrotic tissue when stained by Masson’s trichrome, slices from the liver of the normal group showed no fibrosis. However, excess blue collagen fibers were observed in the experiment group. After treatment with CGA at different concentrations, the area of collagen fibers decreased obviously and high concentration of CGA showed stronger effect of anti-liver fibrosis.

### Effect of CGA on the Protein Expression of α-SMA and Collagen I in Liver Tissue by IHC

As shown in **Figures 9C,D**, the staining of α-SMA and collagen I in the normal group was not remarkable (*P* < 0.01). After induction with CCl4, the positive staining of α-SMA and collagen I in the experimental group significantly increased, showing as dark brown. The staining in the CGA groups showed a smaller area and weaker staining, and fewer positive cells were observed compared with the experimental groups (*P* < 0.05 or 0.01). The inhibitory effect was strengthened when the concentration of CGA increased (*P* < 0.05 or 0.01).

### Evaluation of CGA on the miR-21-Regulated TGF-β1/Smad7 Signaling Pathway in CCl4-Induced Rats

**FIGURE 10:**
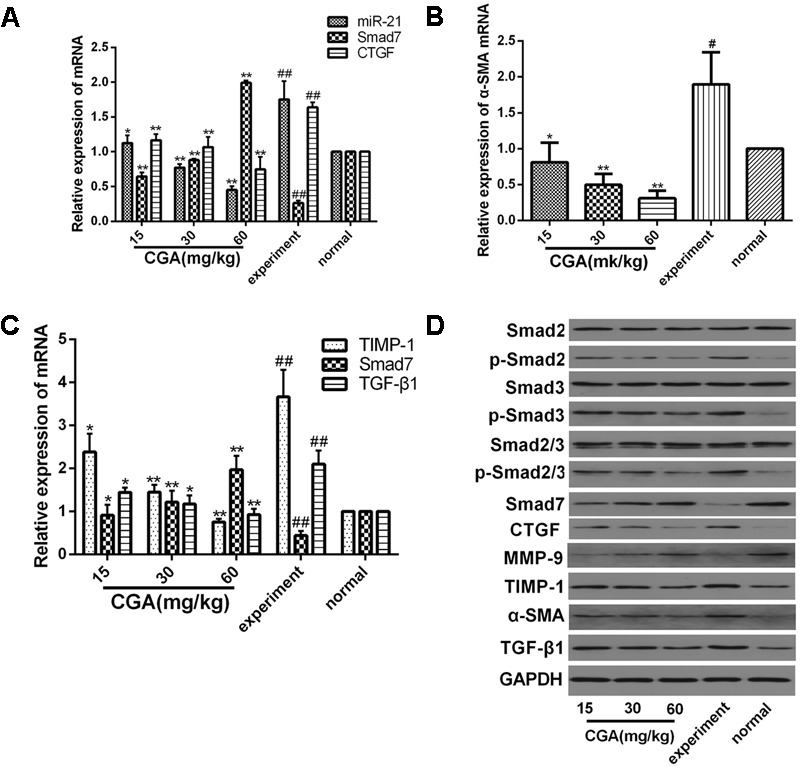
Effect of CGA on the TGF-β1/miR-21/Smad7 signaling pathway in CCl4-induced rats. **(A–C)** The mRNA levels were measured by real-time quantitative PCR. **(D)** The protein levels were assayed by western blotting. The data from three independent experiments are expressed as the means ± SD. ^∗^*P* < 0.05 compared with the experimental group; ^∗∗^*P* < 0.01 compared with experimental group; ^#^*P* < 0.05 compared with the normal group; ^##^*P* < 0.01 compared with the normal group.

As shown in **Figures 10A–C**, compared with the normal group, the mRNA levels of miR-21, CTGF, α-SMA, TIMP-1, and TGF-β1 in the experimental group were markedly increased (*P* < 0.01 or 0.05), and the mRNA levels of Smad7 and MMP-9 was markedly decreased (*P* < 0.01). However, treatment with CGA at different concentrations decreased the mRNA levels of miR-21, CTGF, α-SMA, TIMP-1, and TGF-β1 and elevated the mRNA levels of Smad7 and MMP-9 compared with that in the experimental group (*P* < 0.05 or 0.01). As shown in **Figure 10D**, western blot analysis was carried out to determine the protein expression. Compared with the normal group, the protein expression of p-Smad2, p-Smad3, p-Smad2/3, CTGF, TGF-β1, TIMP-1 and α-SMA were significantly increased, and the protein levels of Smad7 and MMP-9 were decreased in the experimental group. However, the protein expression of p-Smad2, p-Smad3, p-Smad2/3, CTGF, TGF-β1, TIMP-1 and α-SMA was significantly inhibited after CGA treatment, and CGA could elevate Smad7 and MMP-9 expression compared with the experimental group (*P* < 0.05 or 0.01). There were no changes in the protein levels of Smad2, Smad3 and Smad2/3.

## Discussion

Previous studies have shown that liver fibrosis is involved in the regulation of the deposition of ECM by a complex network of signaling pathways, and we have investigated CGA could regulate liver firbosis through IL-13/miR-21/Smad7 signaling way. However, the TGF-β1/Smad signaling pathway is considered another important prominent mediator in promoting liver fibrosis (Bataller and Brenner, 2005; You et al., 2016) and it is one of the most important fibrogenic stimulators. TGF-β1 has many cellular sources, including Kupffer cells, platelets, endothelial cells and lymphocytes (Cui et al., 2010; Hernandez-Gea and Friedman, 2011), and autocrine expression of activated HSC is another important sources (Bartley et al., 2006). TGF-β signaling is initiated by ligand binding to TβR-II, which leads to the activated type II receptor; then, protease TβR-I is phosphorylated, and the TβR-I kinase activates Smad2 and Smad3. Phosphorylated Smad2 and Smad3 then form a complex that translocates to the nucleus and regulates the expression of miR-21, which then prevents Smad7 from regulating Smad2/3 activation through negative feedback by interfering with the target gene of miR-21 and Smad7 (Inagaki and Okazaki, 2007). Meanwhile, a variety of collagens, including a large number of smooth muscle actins (e.g., α-smooth muscle actin and α-SMA), collagen I, collagen III and other ECM components are expressed. Therefore, interfering with the miR-21-regulated TGF-β1/Smad7 signaling pathway may be another effective method to block the development of liver fibrosis.

Connective tissue growth factor (CTGF), synthesized by HSCs and hepatocytes, plays a critical role in the process of liver fibrosis (Huang and Brigstock, 2012). Although the production of CTGF is usually low in normal, healthy liver, increased expression of CTGF was observed in fibrotic livers of both patients and experimental animal models (Hayashi et al., 2002; Kodama et al., 2011). It has been proven CTGF could promote the increase of ECM (Chen et al., 2009). Matrix metalloproteinases (MMPs) and tissue inhibitors of metalloproteinases (TIMPs) are the key factors of the degradation and remodeling of the ECM. MMP-9 is one of the most relevant MMPs that degrades normal liver matrix, and it could promote the development of liver fibrosis (Roderfeld et al., 2006). TIMP1, which has been demonstrated to reduce MMP activity, plays an important role in the progress of liver fibrosis and it is an important target for the treatment of liver fibrosis (Liu et al., 2013).

MicroRNAs (miRNAs) are a group of non-coding small RNA molecules that affect gene expression by binding to the 3′-untranslated region (3′-UTR) of target mRNAs (Ambros, 2004). The development of many diseases, including liver fibrosis, is caused by dysregulation of miR-21 (Png et al., 2011). A high expression of miR-21 has been found in many fibrotic tissues, including the rat liver and human liver (Marquez et al., 2010; Wei et al., 2013). Based on the above studies, we guessed that miR-21 could regulate the development of hepatic fibrosis in LX2 cells, and we up- or down-regulated miR-21 by lentiviral transfection to investigate the effect and specific mechanism of CGA.

In this study, we found that the mRNA levels of miR-21, CTGF, α-SMA, TIMP-1 and TGF-β1 and the protein expression of p-Smad2, p-Smad3, p-Smad2/3, CTGF, TIMP-1, α-SMA, and TGF-β1 in the experimental group were significantly increased compared with that in the normal group, and the mRNA levels of Smad7 and MMP-9 and the protein expression of Smad7 and MMP-9 in the experimental group were significantly decreased compared with those in the normal group. There were no significant changes in the protein levels of Smad2, Smad3 and Smad2/3. After treatment with CGA, the mRNA levels of miR-21, CTGF, α-SMA, TIMP-1, and TGF-β1 and the protein expression of p-Smad2, p-Smad3, p-Smad2/3, CTGF, α-SMA, TIMP-1 and TGF-β1 were inhibited, and the mRNA expression of Smad7 and MMP-9 and the protein expression of Smad7 and MMP-9 were elevated. Meanwhile, TGF-β1 could activate TGF-β1/Smad7 signaling pathway no matter miR-21 was up-regulated or down-regulated in LX2 cells. When treated with CGA in miR-21 up-regulated or down-regulated LX2 cells, the TGF-β1/Smad7 signaling pathway in LX2 cells was significantly inhibited. In addition, in the CCl4-induced fibrosis rat model, compared with the normal group, the level of TGF-β1 in the serum was significantly increased in the experimental group, and CGA could decrease the concentration of TGF-β1 in the serum. CGA could also reduce the expression of α-SMA and collagen I in liver tissue and relieve the degree of liver fibrosis in the pathological manifestation.

## Conclusion

**FIGURE 11:**
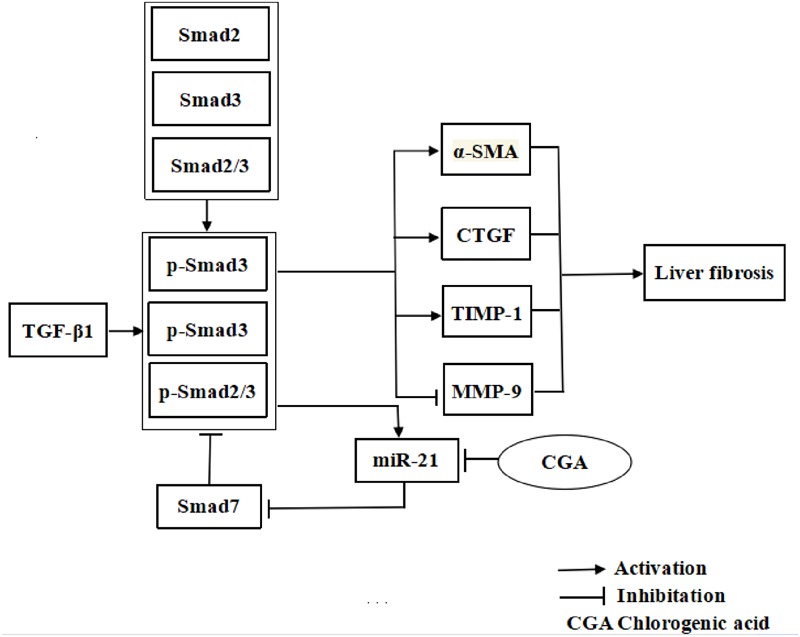
The illustration of CGA protecting against liver fibrosis *in vitro* and *in vivo* by regulating miR-21-regulated TGF-β1/Smad7 signaling pathway.

Chlorogenic acid exerts the ability to suppress liver fibrosis through regulation of the miR-21-regulated TGF-β1/Smad7 signaling pathway *in vivo* and *in vitro* (**Figure 11**), which suggests that CGA may be an attractive anti-liver fibrosis agent. However, it is important to explore whether CGA targets the anti-liver fibrosis effect through other than the TGF-β1 pathway. We are hoping that additional studies on the anti-liver fibrosis mechanisms of CGA may give us a more complete understanding so that new methods to prevent and treat liver fibrosis can be identified.

## Author Contributions

LZ conceived the study. FY, LL, and Z-DZ designed the experiments. LL, YW, XZ, Z-LC, and PL performed the most experiments. XC, Y-FC, and Y-JW performed the feeding of animal and the evaluation of animal histopathology. JX, JZ, QM, and Y-YL analyzed data. LL wrote the manuscript. All authors reviewed the manuscript.

## Conflict of Interest Statement

The authors declare that the research was conducted in the absence of any commercial or financial relationships that could be construed as a potential conflict of interest. The reviewer XT and handling editor declared their shared affiliation.

## References

[B1] Ambros V. (2004). The functions of animal microRNAs. *Nature* 43 350–355. 10.1038/nature02871 15372042

[B2] Bai G. Yan G. Wang G. Wan P. Zhang R. (2016). Anti-hepatic fibrosis effects of a novel turtle shell decoction by inhibiting hepatic stellate cell proliferation and blocking TGF-β1/Smad signaling pathway in rats. *Oncol. Rep.* 36 2902–2910. 10.3892/or.2016.5078 27633729

[B3] Bartley P. B. Ramm G. A. Jones M. K. Ruddell R. G. Li Y. McManus D. P. (2006). A contributory role for activated hepatic stellate cells in the dynamics of *Schistosoma japonicum* egg-induced fibrosis. *Int. J. Parasitol.* 36 993–1001. 10.1016/j.ijpara.2006.04.015 16806222

[B4] Bataller R. Brenner D. A. (2005). Liver fibrosis. *J. Clin. Investig.* 115 209–218. 10.1172/JCI24282 15690074 PMC546435

[B5] Bhattacharyya S. Majhi S. Saha B. P. Mukherjee P. K. (2014). Chlorogenic acid–phospholipid complex improve protection against UVA induced oxidative stress. *J. Photochem. Photobiol. B* 130 293–298. 10.1016/j.jphotobiol.2013.11.020 24378330

[B6] Chen X. M. Qi W. Pollock C. A. (2009). CTGF and chronic kidney fibrosis. *Front. Biosci.* 1 132–141. 10.2741/s13 19482689

[B7] Clifford M. N. (1999). Chlorogenic acids and other cinnamates—nature, occurrence and dietary burden. *J. Sci. Food Agric.* 79 362–372. 10.1002/(SICI)1097-0010(19990301)79:3<362::AID-JSFA256>3.0.CO;2-D

[B8] Cui W. Jin H. B. Li Z. W. (2010). Mechanism of the transforming growth factor-beta induction of fibronectin expression in hepatic stem-like cells. *Braz. J. Med. Biol. Res.* 43 36–42. 10.1590/S0100-879X2009007500017 19936542

[B9] Ding Y. Li G. Xiong L. J. Yin W. Liu J. Liu F. (2015). Profiles of responses of immunological factors to different subtypes of Kawasaki disease. *BMC Musculoskelet. Disord.* 16:315. 10.1186/s12891-015-0744-6 26497060 PMC4619387

[B10] Ding Y. Xiong X. L. Zhou L. S. Yan S. Q. Qin H. Li H. R. (2016). Preliminary study on Emodin alleviating alpha-naphthylisothiocyanate-induced intrahepatic cholestasis by regulation of liver farnesoid X receptor pathway. *Int. J. Immunopathol. Pharmacol.* 29 805–811. 10.1177/0394632016672218 27707957 PMC5806847

[B11] Du P. Ma Q. Zhu Z. D. Li G. Wang Y. Li Q. Q. (2016). Mechanism of Corilagin interference with IL-13/STAT6 signaling pathways in hepatic alternative activation macrophages in schistosomiasis-induced liver fibrosis in mouse model. *Eur. J. Pharmacol.* 793 119–126. 10.1016/j.ejphar.2016.11.018 27845069

[B12] Friedman S. L. (2003). Liver fibrosis—from bench to bedside. *J. Hepatol.* 38(Suppl. 1) S38–S53. 10.1016/S0168-8278(02)00429-4 12591185

[B13] Gu L. Tao X. Xu Y. Han X. Qi Y. Xu L. (2016). Dioscin alleviates BDL- and DMN-induced hepatic fibrosis via Sirt1/Nrf2-mediated inhibition ofp38 MAPK pathway. *Toxicol. Appl. Pharmacol.* 292 19–29. 10.1016/j.taap.2015.12.024 26747300

[B14] Guo Y. J. Luo T. Wu F. Liu H. Li H. R. Mei Y. W. (2015a). Corilagin protects against HSV1 encephalitis through inhibiting the TLR2 signaling pathways in vivo and in vitro. *Mol. Neurobiol.* 52 1547–1560. 10.1007/s12035-014-8947-7 25367881

[B15] Guo Y. J. Luo T. Wu F. Mei Y. W. Peng J. Liu H. (2015b). Involvement of TLR2 and TLR9 in the anti-inflammatory effects of chlorogenic acid in HSV-1-infected microglia. *Life Sci.* 127 12–18. 10.1016/j.lfs.2015.01.036 25744394

[B16] Hayashi N. Kakimuma T. Soma Y. Grotendorst G. R. Tamaki K. Harada M. (2002). Connective tissue growth factor is directly related to liver fibrosis. *Hepatogastroenterology* 49 133–135. 11941937

[B17] Hernandez-Gea V. Friedman S. L. (2011). Pathogenesis of liver fibrosis. *Annu. Rev. Pathol.* 6 425–456. 10.1146/annurev-pathol-011110-130246 21073339

[B18] Huang G. Brigstock D. R. (2012). Regulation of hepatic stellate cells by connective tissue growth factor. *Front. Biosci.* 17 2495–2507. 10.2741/4067 22652794

[B19] Huang Y. F. Zhang S. L. Jin F. Cheng D. Zhou Y. P. Li H. R. (2013). Activity of corilagin on post-parasiticide liver fibrosis in Schistosomiasis animal model. *Int. J. Immunopathol. Pharmacol.* 26 85–92. 10.1177/03946320130260108 23527711

[B20] Ikushima H. Miyazono K. (2012). TGF-β signal transduction spreading to a wider field: a broad variety of mechanisms for context-dependent effects of TGF-β. *Cell Tissue Res.* 347 37–49. 10.1007/s00441-011-1179-5 21618142

[B21] Inagaki Y. Okazaki I. (2007). Emerging insights into transforming growth factor beta Smad signal in hepatic fibrogenesis. *Gut* 56 284–292. 10.1136/gut.2005.088690 17303605 PMC1856752

[B22] Iredale J. P. (2007). Models of liver fibrosis: exploring the dynamic nature of inflammation and repair in a solid organ. *J. Clin. Invest.* 117 539–548. 10.1172/JCI30542 17332881 PMC1804370

[B23] Jin F. Cheng D. Tao J. Y. Zhang S. L. Pang R. Guo Y. J. (2013). Anti-inflammatory and anti-oxidative effects of corilagin in a rat model of acute cholestasis. *BMC Gastroenterol.* 13:79. 10.1186/1471-230X-13-79 23641818 PMC3655894

[B24] Jin F. Zhang R. Feng S. Yuan C. T. Zhang R. Y. Han G. K. (2015). Pathological features of transplanted tumor established by CD133 positive TJ905 glioblastoma. *Cancer Cell Int.* 15:60. 10.1186/s12935-015-0208-y 26136642 PMC4487198

[B25] Kodama T. Takehara T. Hikita H. Shimizu S. Shigekawa M. Tsunematsu H. (2011). Increases in p53 expression induce CTGF synthesis by mouse and human hepatocytes and result in liver fibrosis in mice. *J. Clin. Invest.* 121 3343–3356. 10.1172/JCI44957 21747166 PMC3148730

[B26] Li G. H. Wang X. Xu Y. F. Zhang B. G. Xia X. D. (2014). Antimicrobial effect and mode of action of chlorogenic acid on *Staphylococcus aureus*. *Eur. Food Res. Technol.* 238 589–596. 10.1007/s00217-013-2140-5

[B27] Li H. R. Li G. Li M. Zhang S. L. Wang H. Luo T. (2016). Corilagin ameliorates schistosomiasis hepatic fibrosis through regulating IL-13 associated signal pathway in vitro and in vivo. *Parasitology* 143 1629–1638. 10.1017/S0031182016001128 27439782

[B28] Li H. R. Liu J. Zhang S. L. Luo T. Wu F. Dong J. H. (2017). Corilagin ameliorates the extreme inflammatory status in sepsis through TLR4 signaling pathways. *BMC Complement. Altern. Med.* 17:18. 10.1186/s12906-016-1533-y 28056977 PMC5217594

[B29] Liu J. Cheng X. Guo Z. Wang Z. Li D. Kang F. (2013). Truncated active human matrix metalloproteinase-8 delivered by a chimeric adenovirus-hepatitis B virus vector ameliorates rat liver cirrhosis. *PLOS ONE* 8:e53392. 10.1371/jour-nal.pone.0053392 23301066 PMC3536652

[B30] Luedde T. Schwabe R. F. (2011). NF-κB in the liver—linking injury, fibrosis and hepatocellular carcinoma. *Nat. Rev. Gastroenterol. Hepatol.* 8 108–118. 10.1038/nrgastro.2010.213 21293511 PMC3295539

[B31] Luo H. J. Wang J. Z. Chen J. F. Chen J. F. Zou K. (2011). Docking study on chlorogenic acid as a potential H5N1 influenza A virus neuraminidase inhibitor. *Med. Chem. Res.* 20 554–557. 10.1007/s00044-010-9336-z

[B32] Marquez R. T. Bandyopadhyay S. Wendlandt E. B. Keck K. Hoffer B. A. Icardi M. S. (2010). Correlation between microRNA expression levels and clinical parameters associated with chronic hepatitis C viral infection in humans. *Lab. Invest.* 90 1727–1736. 10.1038/labinvest.2010.126 20625373

[B33] Monteiro M. Farah A. Perrone D. Truqo L. C. Donanqelo C. (2007). Chlorogenic acid compounds from coffee are differentially absorbed and metabolized in humans. *J. Nutr.* 137 2196–2201. 17884997 10.1093/jn/137.10.2196

[B34] Niggeweg R. Michael A. J. Martin C. (2004). Engineering plants with increased levels of the antioxidant chlorogenic acid. *Nat. Biotechnol.* 22 746–754. 10.1038/nbt966 15107863

[B35] Onakpoya I. J. Spencer E. A. Thompson M. J. Heneghan C. J. (2015). The effect of chlorogenic acid on blood pressure: a systematic review and meta-analysis of randomized clinical trials. *J. Hum. Hypertens.* 29 77–81. 10.1038/jhh.2014.46 24943289

[B36] Png K. J. Halberg N. Yoshida M. Tavazoie S. F. (2011). A microRNA regulon that mediates endothelial recruitment and metastasis by cancer cells. *Nature* 481 190–194. 10.1038/nature1066 22170610

[B37] Roderfeld M. Weiskirchen R. Wagner S. Berres M. L. Henkel C. Grötzinger J. (2006). Inhibition of hepatic fibrogenesis by matrix metalloproteinase-9 mutants in mice. *FASEB J.* 20 444–454. 10.1096/fj.05-4828com 16507762

[B38] Saito D. Kyakumoto S. Chosa N. Ibi M. Takahashi N. Okubo N. (2013). Transforming growth factor-β1 induces epithelial-mesenchymal transition and integrin α3β1-mediated cell migration of HSC-4 human squamous cell carcinoma cells through Slug. *J. Biochem.* 153 303–315. 10.1093/jb/mvs144 23248240

[B39] Schuppan D. Kim Y. O. (2013). Evolving therapies for liver fibrosis. *J. Clin. Invest.* 123 1887–1901. 10.1172/JCI66028 23635787 PMC3635731

[B40] Shi H. Shi A. Dong L. Lu X. Wang Y. Zhao J. (2016). Chlorogenic acid protects against liver fibrosis in vivo and in vitro through inhibition of oxidative stress. *Clin. Nutr.* 35 1366–1373. 10.1016/j.clnu.2016.03.002 27017478

[B41] Shin H. S. Satsu H. Bae M. J. Zhao Z. Oqiwara H. Tostuka M. (2015). Anti-inflammatory effect of chlorogenic acid on the IL-8 production in Caco-2 cells and the dextran sulphate sodium-induced colitis symptoms in C57BL/6 mice. *Food Chem.* 168 167–175. 10.1016/j.foodchem.2014.06.100 25172696

[B42] Suzuki A. Yamamoto N. Jokura H. Yamamoto M. Fujii A. Tokimitsu I. (2006). Chlorogenic acid attenuates hypertension and improves endothelial function in spontaneously hypertensive rats. *J. Hypertens.* 24 1065–1073. 10.1097/01.hjh.0000226196.67052.c0 16685206

[B43] Wang Y. Yang F. Xue J. Zhou X. Luo L. Ma Q. (2017). Anti-Schistosomiasis liver fibrosis effects of chlorogenic acid through IL-13/miR-21/Smad7 signaling interactions *in vivo* and *in vitro*. *Antimicrob. Agents Chemother.* 61:e01347–16. 10.1128/AAC.01347-16 27872076 PMC5278737

[B44] Wei J. Feng L. Li Z. Xu G. Fan X. (2013). MicroRNA-21 activates hepatic stellate cells via PTEN/Akt signaling. *Biomed. Pharmacother.* 67 387–392. 10.1016/j.biopha.2013.03.014 23643356

[B45] Wells R. G. (2000). Fibrogenesis V. TGF-beta signaling pathways. *Am. J. Physiol. Gastrointest. Liver Physiol.* 279 G845–G850. 11052979 10.1152/ajpgi.2000.279.5.G845

[B46] Xu T. Ni M. M. Li X. Li X. F. Meng X. M. Huang C. (2016). NLRC5 regulates TGF-β1-induced proliferation and activation of hepatic stellate cells during hepatic fibrosis. *Int. J. Biochem. Cell Biol.* 70 92–104. 10.1016/j.biocel.2015.11.010 26592197

[B47] Xu Y. X. Chen J. W. Yu X. A. Tao W. Jiang F. Yin Z. (2010). Protective effects of chlorogenic acid on acute hepatotoxicity induced by lipopolysaccharide in mice. *Inflamm. Res.* 59 871–877. 10.1007/s00011-010-0199-z 20405164

[B48] Yan L. Meyer C. Müller A. Herweck F. Li Q. Müllenbach R. (2011). IL-13 induces connective tissue growth factor in rat hepatic stellate cells via TGF-β-independent Smad signaling. *J. Immunol.* 187 2814–2823. 10.4049/jimmunol.1003260 21804025

[B49] Yang F. Wang Y. Xue J. Ma Q. Zhang J. Chen Y. F. (2016). Effect of Corilagin on miR-21/smad7/ERK signal pathway in schistosomiasis-induced hepatic fibrosis in mouse model. *Parasitol. Int.* 65 308–315. 10.1016/j.parint.2016.03.001 26946098

[B50] You S. P. Ma L. Zhao J. Zhang S. L. Liu T. (2016). Phenylethanol glycosides from *Cistanche tubulosa* suppress hepatic stellate cell activation and block the conduction of signaling pathways in TGF-β1/smad as potential anti-hepatic fibrosis agents. *Molecules* 21:102. 10.3390/molecules21010102 26797590 PMC6273390

[B51] Zhang X. Han X. Yin L. Xu L. Qi Y. Xu Y. (2015a). Potent effects of dioscin against liver fibrosis. *Sci. Rep.* 5:9713. 10.1038/srep09713 25853178 PMC4389718

[B52] Zhang X. Xu L. Yin L. Qi Y. Xu Y. Han X. (2015b). Quantitative chemical proteomics for investigating the biomarkers of dioscin against liver fibrosis caused by CCl4 in rats. *Chem. Commun.* 51 11064–11067. 10.1039/c4cc09160d 26069897

[B53] Zhou Y. P. Zhang S. L. Cheng D. Li H. R. Tang Z. M. Xue J. (2013). Preliminary exploration on anti-fibrosis effect of kaempferol in mice with japonicum schistosoma infection. *Eur. J. Inflamm.* 11 161–168. 10.1177/1721727X1301100115

